# Crack growth pattern analysis of monolithic glass ceramic on a titanium abutment for single crown implant restorations using smooth particle hydrodynamics algorithm

**DOI:** 10.34172/japid.2021.005

**Published:** 2021-04-20

**Authors:** Mohammad Kashfi, Parisa Fakhri, Ataollah Ghavamian, Payam Pourrabia, Fatemeh Salehi Ghalesefid, Parviz Kahhal

**Affiliations:** ^1^Mechanical Engineering Department, Ayatollah Boroujerdi University, Boroujerd, Iran; ^2^Instrumentation Research Group, Niroo Research Institute (NRI), Tehran, Iran; ^3^Senior R & D Engineer, FEops, Ghent, Belgium; ^4^Department of Prosthodontics, School of Dentistry, Isfahan University of Medical Sciences, Isfahan, Iran

**Keywords:** Crack propagation, Mechanical properties, Monolithic crowns, Numerical simulation, Smooth particle, Hydrodynamics

## Abstract

**Background:**

Glass ceramic materials have multiple applications in various prosthetic fields. Despite the many advantages of these materials, they still have limitations such as fragility and surface machining and ease of repairing. Crack propagation has been a typical concern in fullceramic crowns, for which many successful numerical simulations have been carried out using the extended finite element method (XFEM). However, XFEM cannot correctly predict a primary crack growth direction under dynamic loading on the implant crown.

**Methods:**

In this work, the dental implant crown and abutment were modeled in CATIA V5R19 software using a CT-scan technique based on the human first molar. The crown was approximated with 39514 spherical particles to reach a reasonable convergence in the results. In the present work, glass ceramic was considered the crown material on a titanium abutment. The simulation was performed for an impactor with an initial velocity of 25 m/s in the implant-abutment axis direction. We took advantage of smooth particle hydrodynamics (SPH) such that the burden of defining a primary crack growth direction was suppressed.

**Results:**

The simulation results demonstrated that the micro-crack onset due to the impact wave in the ceramic crown first began from the crown incisal edge and then extended to the margin due to increased stress concentration near the contact region. At 23.36 µs, the crack growth was observed in two different directions based on the crown geometry, and at the end of the simulation, some micro-cracks were also initiated from the crown margin. Moreover, the results showed that the SPH algorithm could be considered an alternative robust tool to predict crack propagation in brittle materials, particularly for the implant crown under dynamic loading.

**Conclusion:**

The main achievement of the present study was that the SPH algorithm is a helpful tool to predict the crack growth pattern in brittle materials, especially for ceramic crowns under dynamic loading. The predicted crack direction showed that the initial crack was divided into two branches after its impact, leading to the crown fracture. The micro-crack initiated from the crown incisal edge and then extended to the crown margin due to the stress concentration near the contact area.

## Introduction

Predictable mechanical efficacy is one of the essential requirements for manufacturing prosthetic crowns. Despite the many advantages of monolithic ceramic crowns, they still have limitations such as fragility and surface machining and ease of repairing.^1,2^ Numerical simulation is a strong tool for predicting the fracture pattern of engineered materials and analyzing their damage behavior.^3-6^ Smooth particle hydrodynamics (SPH) is one of the most robust computational methods employed to simulate solid mechanics^7^ and fluid flows developed by Gingold, Monaghan.^8^ The classical SPH Lagrangian formulation is well-known to suffer from some numerical drawbacks.^9^ However, Lee et al^10^ introduced a total Lagrangian upwind algorithm for large strain explicit solid dynamics to improve the stability and accuracy of the SPH analysis.

Lai et al^11^ investigated the resistance of functionally graded cementitious composite against repeated penetration. The penetration depth, fracture pattern, and penetration damage of different concrete targets have been numerically studied using the SPH method. They found that SPH could predict the dynamic fracture pattern with reasonable accuracy in comparison with the experiment. Based on the literature survey, relevant and reasonable studies on SPH methods to obtain the mechanical behavior of restorative ceramic materials considering these material’s features are lacking. In the present work, crack propagation under dynamic loading of an implant crown was investigated using the SPH method.

## Methods

### The theoretical background of SPH

The SPH method discretizes a continuum into a set of particles. These particles interact through a kernel interpolation function with a characteristic radius known as the “smoothing length” (h). It implies that any particle’s physical properties can be obtained by summing the relevant properties of all the particles that lie within the compact support of the kernel. This can be understood in two steps. First, an arbitrary field A is written as a convolution with W:

(1) $$A\left(r\right)=\int A\left(r^{\prime }\right)\;W\left(\left|r-r^{\prime }\right|,h\right)dV\left(r^{\prime }\right)$$

Secondly, the integral is approximated using a Riemann summation over the particles:

(2) $$A\left(r\right)\approx \sum_{j}V_{j}A_{j}W\left(\left|r-r_{j}\right|,h\right)$$

where the summation over j includes all the particles lying within the compact support of the kernel, V_j_ is the volume, A_j_ is the value of a quantity, and r denotes the position. For example, the density ρ_i_ of particle i can be expressed as:

(3) $$\rho_{i}=\rho \left(r_{i}\right)=\sum_{j}m_{j}W_{ij}$$

Where *m*_j_* =p*_j_*V*_i_ denotes the particle mass, and ρ_i_ is the particle density and *W*_ij_* = W*_ji_ is a short notation for.*W*(*|r-r*_j_*|,h*) The quality of SPH approximation relies on different characteristics such as smoothing length, particle distribution, and particle size, to name a few. In this work, we considered a constant smoothing length and regular particle distribution.^12^

### Problem geometry

**Figure 1:**
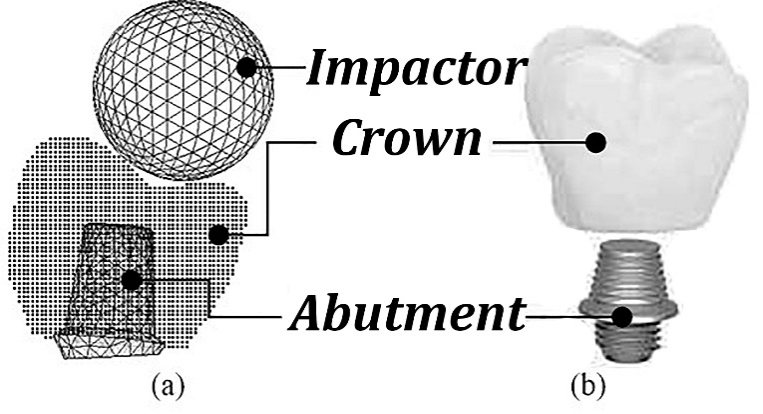
The geometry of the implant parts.

The geometry used in the present study is shown in Figure 1 (a). The dental implant crown and abutment were modeled in CATIA V5R19 software using a CT-scan technique based on the human first molar.^13^

### SHP model construction

AUTODYN explicit hydrodynamic code was employed to construct the numerical model for predicting the implant crown fracture pattern based on the SPH formulation. The numerical model is depicted in Figure 1 (b). The impactor was also constructed as a sphere using Lagrangian elements. The crown was approximated with 39514 spherical particles to reach a reasonable convergence in the results. 3D Lagrangian elements also construct the abutment numerical model. In the present work, glass ceramic was considered the crown material on a titanium abutment. The linear isotropic material model was assumed based on the nature of the materials. The frictionless Lagrangian/Lagrangian contact method with automatic gap detector was considered the interaction algorithm between the simulated abutment and crown.

Moreover, self-interaction was activated for the crown. The material model constants for all parts were assigned to the numerical model as reported in reference.^14^ The simulation was performed for an impactor with 25 m/s initial velocity in the implant-abutment axis direction. The base region of the abutment was fixed in all directions as the model boundary condition.

## Results

**Figure 2:**
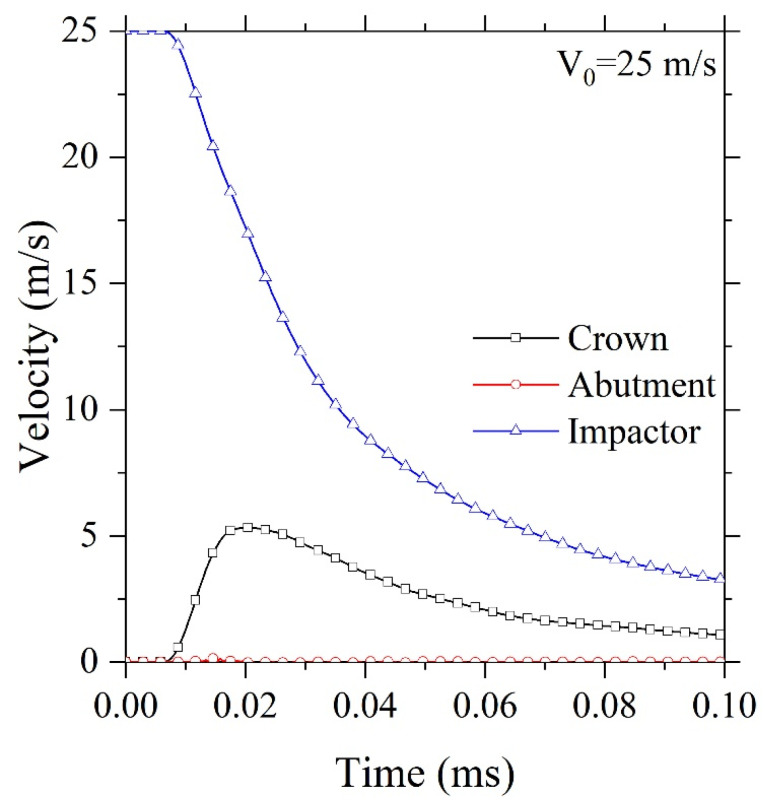
Velocity versus time predicted by numerical simulation for the crown, abutment, and impactor.

Figure 2 shows the predicted velocity of the impactor and crown during the dynamic impact loading. As it can be seen, the impactor velocity was reduced from 25 m/s to 3 m/s within 0.1 ms. However, the induced velocity in the crown is increased to 5 m/s within 0.02 m/s and then decreased to 1 m/s at the end of the simulation. The velocity of implant-abutment remained near zero during the impact.

**Figure 3:**
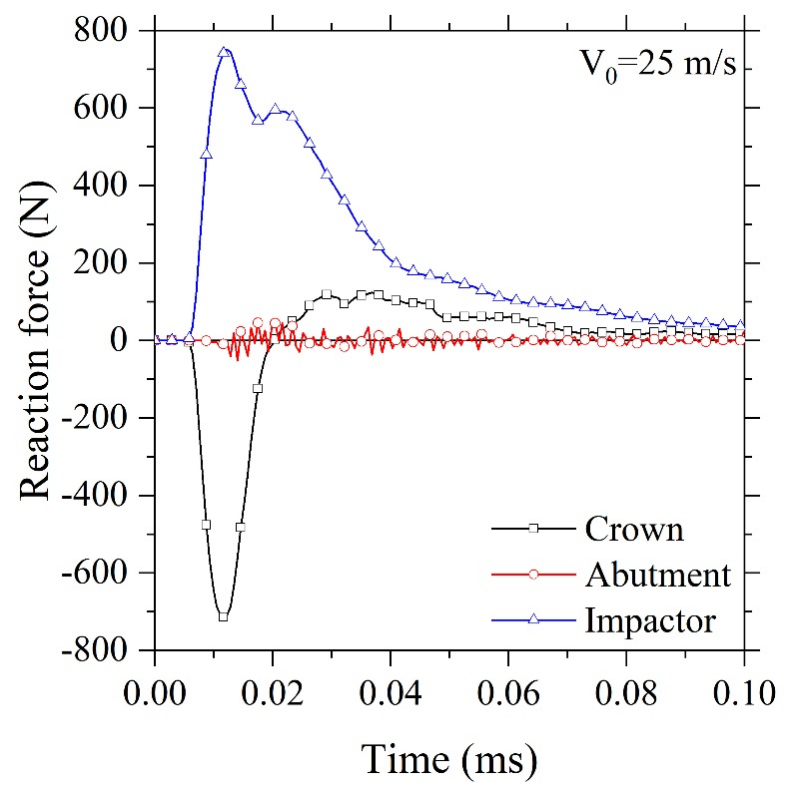
Reaction force versus time predicted by numerical simulation for the crown, abutment, and impactor.

Figure 3 shows the predicted reaction force during the numerical simulation. It is worth noting that the reaction force was measured based on the body forces during the body contact. The maximum force was applied to the crown at about 0.01 ms, and it was then suddenly decreased. In contrast, the reaction force of the impactor was increased while the two bodies were touching. The measured impactor reaction force was then reduced due to the crack propagation.

The induced reaction force of the impactor was decreased due to the dissipated energy during the crack initiation and crack propagation. Up to 0.1 ms, all reaction forces were converged to zero with reasonable accuracy.

**Figure 4:**
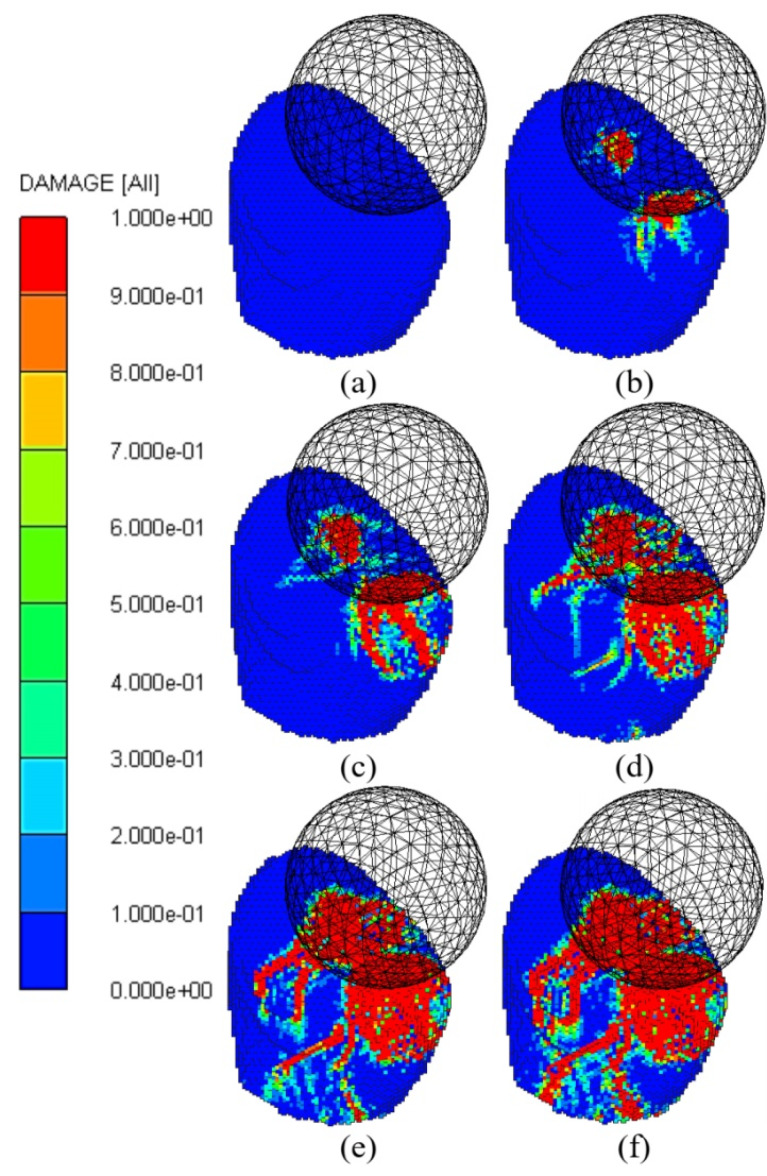
Damage evolution contour in crown during impact time (a) 0 ms, (b) 1.167E-2 ms, (c) 1.752E-2 ms, (d) 2.336E-2 ms, (e) 2.920E-2 ms and (f) 3.505E-2 ms.

Figure 4 illustrates the evolution of the damage contour in the crown during the impact simulation. At 1.167E-2 ms, the impactor touched the incisal edge of the crown. The red color in the damage contour indicated the region in which the crown was entirely eroded during the dynamic loading. The micro-cracks initiated from the incisal edge of the crown and grew over time. As shown in Figure 4 (d), the simulated micro-cracks extended to visible micro-cracks in the crown, leading to fracturing.

In the SPH method, there was no need to define the crack path. This is one of the most important benefits of the SPH method compared with FEM. At 2.336E-2 ms, the crack growth was observed in two different directions based on the crown geometry. As Figure 4 suggests, some micro-cracks also initiated from the crown base region at the end of the simulation.

## Discussion

**Figure 5:**
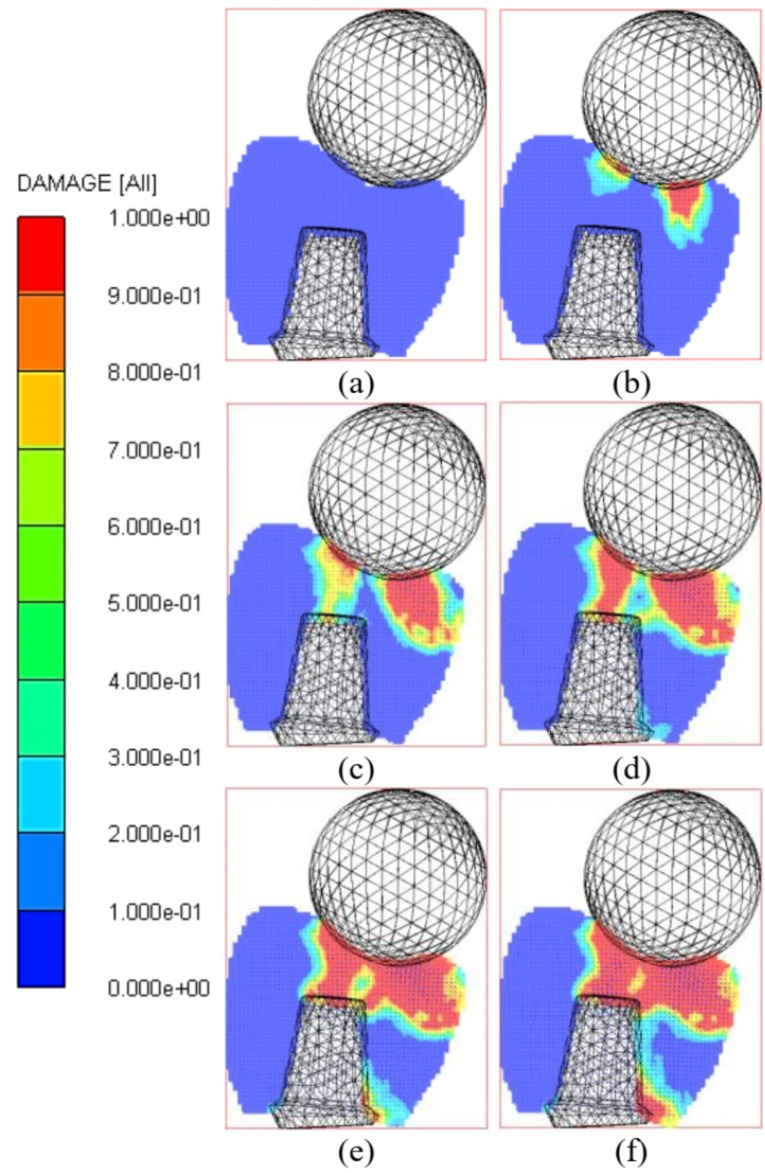
Damage evolution contour in the crown lateral cross-section during the impact time (a) 0 ms, (b) 1.167E-2 ms, (c) 1.752E-2 ms, (d) 2.336E-2 ms, (e) 2.920E-2 ms and (f) 3.505E-2 ms.

Ceramic restorations have become popular because of their favorable mechanical properties, excellent biocompatibility, and pleasing esthetics. However, they are physically weak, and the stress state applied in the oral cavity is complex. It is desirable to evaluate the loads a restoration must endure during function over time. It is more appropriate to assess analogous tooth-shaped restorations under conditions similar to the oral cavity.^15^ Studies on the methods to obtain the mechanical behavior of restorative ceramic materials considering the features of these materials are lacking. Hence in this study, the SPH method has been employed to investigate the fracture pattern during material cracking. For a better presentation of the fracture pattern in the implant-supported single crown, a lateral cross-section is provided in Figure 5. As the figure indicates, the crack reached the crown cavity where the implant abutment was placed at 1.752E-2 ms, implying that the crown had lost its stiffness significantly, making it more prone to fracture.

As Figure 5 clearly shows, the crack started from 2.336E-2 ms. The residual velocity of the impactor in the present time was determined about 15 m/s, indicating that with up to 10 m/s reductions in speed, the full-ceramic crown has resisted crack initiation close to the abutment. Moreover, the crown is fully damaged up to 3 m/s and could no longer bear the load. The crown undergoing the imposed dynamic load could resist a maximum velocity of 5 m/s, as depicted in Figure 2 at 1.752E-2 ms.

Moreover, as seen in Figure 5 (f), the crack growth rate of the base region on the abutment increased due to its geometry and stress concentration. It is recommended to redesign the implant abutment by using a round fillet at the end of the abutment, which probably takes several numerical simulations and optimizations. The fillet radius could be considered the design variable, and the crack growth rate might be assumed as the objective function that should be minimized.

The main limitation of the present work is the variability of the crown geometry per person/patient. Moreover, the effect of temperature change is an interesting topic that could be studied in the future by coupling mechanical and thermal material properties. Besides, although feldspathic porcelain has been shown to have a low strength compared with other commonly used materials in dental restoration, in this research, the ability of SPH to predict the crack growth pattern of a feldspathic crown as a case study under the dynamic loading has been investigated. We hope that the present study opens up new horizons to prove the ability of the SPH algorithm to predict the fracture pattern of the single-crown implant restorations. Since an accurate SPH simulation requires several mechanical properties supported by particular material characterization experiments, it is suggested for future studies to conduct high-velocity experiments on the recently common materials, such as monolithic zirconia or zirconia layered with porcelain.

## Conclusion

In the present paper, the crack growth pattern of ceramic crowns undergoing dynamic loading was investigated using the SPH algorithm. The main achievements of the present study could be summarized as follows:

The SPH algorithm is a useful tool to predict the crack growth pattern in the brittle material, especially for ceramic crowns under dynamic loading.

The predicted crack direction shows that the initial crack is divided into two branches after the impactor’s impact, leading to crown fracture.

The simulation results demonstrated that the micro-crack based on the impact wave in the ceramic crowns initiated from the crown incisal edge and then extended to the crown margin due to the stress concentration near the contact region.

## Authors Contributions

MK: Conceptualization, writing the original draft, simulation review, and editing. PF: Review, literature review, and editing. AG: Review, SHP reviewing, and English editing. PP: Simulation, manuscript preparation, and literature review. FSG: Review, literature review, and editing. PK: Review and editing.

## Ethical approval

No humans participated in this study. The study did not have any ethical registrations.

## Competing interests

The authors declare that they have no competing interests related to authorship and/or publication of this work.
